# AMPK in the Ventromedial Nucleus of the Hypothalamus: A Key Regulator for Thermogenesis

**DOI:** 10.3389/fendo.2020.578830

**Published:** 2020-09-23

**Authors:** Hailan Liu, Yong Xu, Fang Hu

**Affiliations:** ^1^Department of Metabolism and Endocrinology, Metabolic Syndrome Research Center, National Clinical Research Center for Metabolic Diseases, The Second Xiangya Hospital of Central South University, Changsha, China; ^2^Department of Pediatrics, Children's Nutrition Research Center, Baylor College of Medicine, Houston, TX, United States; ^3^Department of Molecular and Cellular Biology, Baylor College of Medicine, Houston, TX, United States

**Keywords:** AMPK, VMH, SNS, thermogenesis, obesity

## Abstract

Obesity has become a global health issue, but effective therapies remain very limited. Adaptive thermogenesis promotes weight loss by dissipating energy in the form of heat, thereby representing a promising target to counteract obesity. Notably, the regulation of thermogenesis is tightly orchestrated by complex neuronal networks, especially those in the hypothalamus. Recent evidence highlights the importance of adenosine monophosphate-activated protein kinase (AMPK) within the ventromedial nucleus of the hypothalamus (VMH) in modulating thermogenesis. Various molecules, such as GLP-1, leptin, estradiol, and thyroid hormones, have been reported to act on the VMH to inhibit AMPK, which subsequently increases thermogenesis through the activation of the sympathetic nervous system (SNS). In this review, we summarize the critical role of AMPK within the VMH in the control of energy balance, focusing on its contribution to thermogenesis and the associated mechanisms.

## Introduction

Obesity and its related metabolic disorders, including type 2 diabetes, cardiovascular diseases and cancer, are major health threats which cause thousands of deaths per year in the contemporary society (1). Given the current obesity epidemic, there is a pressing need for novel therapeutic interventions to help people manage their body weight more efficiently (1). Owing to their ability to trigger thermogenesis and enhance energy utilization, brown and beige adipose tissues have recently been identified as a promising target for obesity (2–4). More importantly, accumulating evidence suggests that brown adipocytes are also present in adult humans and are associated with improved metabolic profiles (5, 6). In this sense, therapies aimed at amplifying the thermogenic capabilities of brown and beige adipocytes are of great translational significance.

At the whole-body level, the hypothalamus plays a crucial role in controlling thermogenesis in brown and beige adipose tissues (7). Several hypothalamic nuclei, including the ventromedial (VMH), arcuate (ARH), dorsomedial (DMH), and paraventricular (PVH) nuclei, as well as the preoptic (POA) and lateral hypothalamic (LHA) areas, have been demonstrated to participate in the regulation of adaptive thermogenesis (8). In particular, the VMH exerts a well-established action on brown adipose tissue (BAT) thermogenesis through its close link with the brainstem areas, including the rostral raphe pallidus (rRPa) and inferior olive (IO), which are involved in modulating BAT function through the SNS (9–11). Electrical stimulation of the VMH increases thermogenesis and BAT temperature, whereas lesions in the VMH inhibit thermogenesis and energy expenditure (12, 13).

As a highly conserved serine/threonine kinase, AMPK integrates peripheral and central metabolic signals to regulate energy homeostasis (14). In addition to its well-established effects on feeding, glucose control and insulin sensitivity, AMPK within the VMH regulates thermogenesis by manipulating the sympathetic firing to BAT and WAT (white adipose tissue) (15–17). For example, deletion of AMPK in the VMH ameliorates diet-induced obesity via exaggerating thermogenesis in BAT and WAT (18). This review intends to provide an insight on several hormonal signals acting on the VMH to control adaptive thermogenesis, with a particular focus on their influences on AMPK and downstream reactions.

## Thermogenic Capacity of Brown and Beige Adipocytes: Under Control of the Hypothalamus

Brown adipocytes, characterized by a great number of mitochondria and multilocular lipid droplets, are regarded as the major contributor to adaptive thermogenesis (4, 19). BAT has abundant expression of uncoupling protein 1 (UCP1), which dissipates the electrochemical proton gradient through a proton leak in the inner mitochondrial membrane, resulting in the uncoupling of oxidative phosphorylation from ATP synthesis to heat production (20). Sympathetic nerve releases norepinephrine (NE) to activate BAT via the widely distributed β3-adrenergic receptors (β3-ARs) in brown adipocytes, triggering lipolysis and thermogenesis (7, 21). Above all, functional brown adipocytes are found in discrete depots in adult humans and can be induced by sympathetic stimulus, such as cold (5, 6, 22). From this perspective, stimulation of BAT could have therapeutic potentials for long-term management of body weight in obese individuals (23).

In recent years, a novel type of adipocytes has been identified, termed beige adipocytes, which express the thermogenic genes characteristic of those typically associated with brown adipocytes (24). Beige adipocytes could be developed from white adipocytes through various stimulus, including cold, β3-AR agonists, and numerous circulating hormones, such as leptin and fibroblast growth factor 21 (25–27). The process through which white adipocytes turned into beige adipocytes is known as browning (28). Distinct from white adipocytes, which have a large lipid droplet for the storage of excess fat and few UCP1 and mitochondria, beige adipocytes own many similarities with brown adipocytes in both structure and function (29). Particularly, the amount of UCP1 and mitochondria is much more abundant in beige adipocytes than that in white adipocytes (29). In addition, beige adipocytes are densely innervated by sympathetic fibers and can be activated by the SNS (30). Furthermore, beige adipocytes share similar properties as brown adipocytes with respect to UCP1-mediated thermogenesis (31). Remarkably, clinical studies have revealed that chronic cold exposure promotes the recruitment of beige adipocytes in humans, which is associated with improved insulin sensitivity, glucose and lipid homeostasis (32–34). In particular, the increased glucose uptake ability and endocrine factors secreted by brown and beige fat are considered to play important roles in ameliorating the metabolic abnormalities in obese individuals (35).

Among numerous brain regions, the hypothalamus receives and integrates hormonal and neuronal signals that relay metabolic status of the body, hence plays a major role in controlling adaptive thermogenesis (36). The SNS mediates the crosstalk between the hypothalamus and adipose tissues (37). Anatomically, the POA, VMH, DMH, ARH, PVH, and LHA have direct or indirect connections with the sympathetic preganglionic neurons in the spinal cord (38). Stimulation of the aforementioned hypothalamic nuclei increases the sympathetic tone to BAT, and tonic inhibition of neurons in many of these areas reduces BAT activity (38). Specifically, the ARH contains proopiomelanocortin (POMC) and agouti-related-peptide (AgRP) expressing neurons, which are key components of the melanocortin system that accounts for the regulation of food intake and adaptive thermogenesis (39). POMC and AgRP neurons orchestrate feeding behavior and thermogenesis mainly by releasing several key neuropeptides or neurotransmitters, like α-melanocyte-stimulating hormone (α-MSH), AgRP, neuropeptide Y (NPY), and γ-aminobutyric acid (γ-GABA) (39, 40). These molecules act on their broadly distributed receptors in the central nervous system (CNS) to affect appetite and SNS-mediated thermogenesis (39, 41, 42). In parallel, the participation of the VMH in thermoregulation has been confirmed by emerging evidence. Ablation of steroidogenic factor-1 (SF-1), a transcription factor expressed exclusively in the VMH within the brain, impairs BAT thermogenesis without altering food intake (43). More remarkably, accumulating data point out that several key homeostatic signals act on the VMH to inhibit AMPK activity, which in turn stimulates BAT thermogenesis and WAT browning through the SNS (44).

## Hormonal Regulation of Thermogenesis Through the VMH: Highlighting the Canonical Role of AMPK

The capacity of brown and beige adipocytes to increase adaptive thermogenesis is predominately governed by the hypothalamus through the regulation of the sympathetic outflows (8). Among various hypothalamic nuclei, the VMH plays a fundamental part in modulating BAT function given the fact that VMH neurons are anatomically linked to the rRPa and IO, which perform well-established actions on BAT thermogenesis through the SNS (10, 11, 45). Intra-VMH administration of glutamate or NE increases the activity of neurons in the rRPa and IO, leading to elevated BAT temperature. However, the increase in BAT temperature is abrogated by prior treatment with sympathetic ganglionic blockers or β-AR antagonists, confirming the functional significance of the SNS in mediating VMH stimulation-induced BAT thermogenesis (46–48). Additionally, mice lacking SF-1 or estrogen receptor α (ERα) in the VMH develop an obese phenotype characterized by significantly decreased UCP1 expression in BAT (43, 49). Peripheral signals, such as thyroid hormones (THs), glucagon-like peptide-1 (GLP-1), estradiol (E2), bone morphogenetic proteins (BMP8B), and leptin, act on the VMH to promote BAT thermogenesis and WAT browning (16, 17). Notably, the involvement of endoplasmic reticulum (ER) stress within the VMH in thermoregulation has been uncovered by increasing evidence. Pharmacological and genetic manipulations that exaggerate ER stress in the VMH impair BAT thermogenesis and accelerate the development obesity (50, 51). On the contrary, alleviating ER stress in the VMH is sufficient to improve BAT function and ameliorate diet-induced obesity (51–53). Taken together, these results indicate that the VMH plays an essential role in the regulation of adaptive thermogenesis.

AMPK, an intracellular energy sensor, is composed of a catalytic subunit, α (α1, α2), and two regulatory subunits, β (β1, β2) and γ (γ1, γ2, γ3) (54, 55). The catalytic activity of AMPK is triggered by the phosphorylation of Thr172 on the α subunit, a process initiated by ATP deprivation and inhibited by nutrient supplementation (56). AMPK can also be activated by several upstream kinases, such as liver kinase B1 (LKB1) and calmodulin-dependent kinase kinases (CaMKKs) (57–59). AMPK activation in the hypothalamus augments food intake and diminishes energy expenditure, whereas its inhibition suppresses appetite and increases energy utilization (7, 60, 61). Nevertheless, it is noteworthy that AMPK within the VMH mediates the thermogenic effects of numerous peripheral signals in a feeding independent manner (12, 62, 63). Many hormones, such as THs, GLP-1, E2, BMP8B, and leptin, amplify the sympathetic tone to BAT and WAT by inhibiting AMPK activity in the VMH, resulting in enhanced thermogenesis and energy dissipation independent on food intake (16, 52, 64–66). In stark contrast, constitutive activation of AMPK within the VMH reverses the thermogenic effects of these molecules without altering feeding behavior, verifying the important role of AMPK within the VMH in orchestrating BAT thermogenesis and WAT browning (62, 67). In the following sections of this review, we will discuss several key circulating hormones that act on the VMH to modulate AMPK activity, which subsequently contributes to the control of thermogenesis in BAT and WAT via the SNS.

### Thyroid Hormones (THs)

THs, including triiodothyronine (T3) and thyroxine (T4), regulate a vast range of physiological activities, including growth, development, metabolism, and energy balance (68). The involvement of THs in energy balance has been clearly demonstrated by the phenomenon that the impairment in thyroid function is often accompanied by alterations in food intake and body weight. Hyperthyroidism is linked to hyperphagia and weight loss, whereas hypothyroidism causes appetite suppressing and weight gain (69). THs were originally thought to exert their effects on energy homeostasis by directly acting on peripheral tissues, such as the brown and white adipose tissues, muscle, heart, and liver (70). However, recent data indicate that THs modulate food intake, energy expenditure and body weight by acting, to a large extent, at the central level (70, 71). In support of this view, the α1, α2, β1, and β2 THs receptor (TR) isoforms were found to be widely distributed in the CNS, with the highest expression levels in metabolically active regions, such as the VMH, ARH, and PVH (72).

The significance of the CNS in mediating the effects of THs on energy balance was firstly confirmed by brain specific TRα1 mutant mice, which had elevated T3 concentrations in the hypothalamus and displayed higher food intake, metabolic rate and BAT thermogenic capability (73). In consistent with this, central injection of T3 increases energy expenditure by stimulating BAT thermogenesis and WAT browning (17, 52, 74). UCP1 plays an essential role in mediating T3-induced increase in energy expenditure, and deletion of UCP1 completely abolishes the thermogenic action of central T3 (15). Notably, inactivation of TR in the VMH of hyperthyroid rats blunts weight loss and decreases the expression of thermogenic markers in BAT without concomitant influences on food intake (74), suggesting that the VMH is a key region mediating the thermogenic effect of THs on BAT. Recently, the critical role of AMPK within the VMH in modulating T3-induced alterations in thermogenesis has been revealed by several studies. First, constitutive activation of AMPK within the VMH abrogates T3-induced weight loss and UCP1 expression in BAT and WAT (52). Second, pharmacological or genetic inactivation of AMPK in the VMH fully recapitulates the thermogenic actions of central T3 on BAT and WAT, and such effects could be abolished by application of the β3-AR antagonists (74). Third, ablation of AMPKα1 in SF-1 neurons mimics the actions of T3 in the VMH by enhancing BAT thermogenesis and WAT browning, which in turn protects mice from diet-induced obesity (18, 52). Remarkably, manipulation of AMPK in the VMH selectively impacts the thermogenic aspect of T3 without affecting food intake (18, 52). In brief, these data indicate that THs act centrally to promote BAT thermogenesis and WAT browning via suppressing AMPK activity in the VMH.

Interestingly, current work has demonstrated that ER stress in the VMH plays a role in THs-induced thermogenesis. Hyperthyroid rats exhibit lower hypothalamic ceramide and ER stress levels, which can be reversed by the activation of AMPK in the VMH (52). In contrast, increasing ceramide levels as well as pharmacologically or genetically inducing ER stress in the VMH blunts the effect of central THs on thermogenesis in BAT and WAT (52). This is in line with previous studies which found that alleviation of ER stress by overexpressing the glucose-regulated protein 78 (GRP78), a major ER chaperone protein, in the VMH is sufficient to ameliorate obesity by facilitating BAT thermogenesis and WAT browning (51). These findings support the notion that THs inhibits AMPK activity in the VMH, resulting in reduced ER stress levels, which in turn promotes BAT thermogenesis and WAT browning.

However, some critical limitations of these studies need to be taken into consideration. For example, given its diffusion property, it is likely that adenovirus-mediated manipulation of AMPK or TR or GRP78 is not restricted to the VMH. The involvement of other nuclei in thermoregulation requires more careful evaluation. VMH-specific drug delivery has the same issue. Furthermore, although deleting AMPKα1 in SF-1 neurons reflects functions of the AMPKα1 isoform in the majority of VMH neurons, the role of AMPKα1 deficiency-induced compensatory changes, such as elevated AMPKα2 levels, warrants further investigation.

Except for adipose tissues, THs act centrally to modulate lipid metabolism in the liver. Intra-VMH injection of T3 promotes hepatic lipid accumulation via c-JunN-terminal kinase 1 (cJNK1)-mediated activation of the vagus nerve innervation to liver, which is under control of AMPK but not ER stress (52). Whether AMPK within the VMH mediates the effects of THs on other organs remains to be determined.

### Glucagon-Like Peptide-1 (GLP-1)

GLP-1 is primarily synthesized and secreted by the intestinal L-cells to increase glucose-induced insulin release and decrease glucagon secretion in response to a nutrient load (75). However, GLP-1 secretion is impaired in patients with T2D and obesity (76, 77). GLP-1 agonists are clinically used drugs for T2D, with additional benefits of weight loss and a low risk of hypoglycemia (78, 79). GLP-1 receptors (GLP-1Rs) are expressed in a broad range of neuronal populations, including in many hypothalamic nuclei crucial for the regulation of energy balance (80, 81). In addition, GLP-1 positive cells were found to be distributed in numerous human brain regions (82). Moreover, circulating GLP-1 and its analogs could be transported to the brain and activate neurons in various areas of the CNS (83). Interestingly, GLP-1 is also produced by a small population of preproglucagon neurons located in the brainstem nucleus of the solitary tract (NTS), which project to the hypothalamus to regulate appetite (84, 85).

Liraglutide, a long-acting GLP-1 analog, improves glucose homeostasis and reduces body weight in obese diabetic patients (79). Apart from its participation in glycemic control, liraglutide acts centrally to lower food intake and increase energy expenditure (16, 86). Central injection of liraglutide promotes weight loss via suppressing appetite and increasing energy dissipation, the latter is associated with enhanced lipolysis in WAT and thermogenesis in BAT (87, 88). Particularly, administrating liraglutide into the ARH, PVH or LH decreases food intake and body weight, but does not alter UCP1 expression in BAT and WAT. In opposite, intra-VMH injection of liraglutide has no significant influence on food intake but elevates UCP1 levels in BAT and WAT, resulting in obvious weight loss (16). Thus, central liraglutide participates in the regulation of food intake and energy expenditure by engaging in different hypothalamic nuclei (16, 89). Furthermore, β3-AR antagonists block central liraglutide-induced elevation of UCP1 in BAT and WAT, indicating that the SNS mediates the actions of liraglutide on BAT thermogenesis and WAT browning (16, 89). More importantly, central delivery of liraglutide decreases AMPK activity in the VMH (16). Pharmacological or genetic activation of AMPK in the VMH abolishes the actions of liraglutide on thermogenesis without corresponding alterations in feeding (16), verifying the importance of AMPK within the VMH in mediating liraglutide-induced thermogenesis. Nevertheless, potential engagement of AMPK in adjacent regions of VMH in thermoregulation should be assessed, given the inherent shortcomings of the studies that were analyzed earlier. The thermogenic effects of liraglutide rely on GLP-1Rs, mice lacking the GLP-1Rs in the CNS fail to show any obvious change in BAT temperature or thermogenic markers after the application of liraglutide (89). However, these mutant mice display a normal thermogenic response to cold exposure (89), indicating that endogenous GLP-1Rs are essential for liraglutide-induced thermogenesis but are dispensable for appropriate thermogenic response to cold.

On top of improving glucose control, liraglutide also reduces body weight in obese individuals (90). The weight reducing effects of liraglutide on human subjects have been confirmed by many clinical studies, although whether the decrease in body weight is linked to increased energy expenditure or not remains elusive (91, 92). Recently, the US Food and Drug Administration (FDA) committee has approved the application of liraglutide as an anti-obesity therapy. Other GLP-1 analogs, such as exendin-4, can act on the hypothalamus to inhibit AMPK, thereby suppressing appetite and body weight (93–95). However, the involvement of exendin-4 in the regulation of energy expenditure and the participation of AMPK within the VMH in this process are not clear, and additional work will be necessary to address these questions.

### Estrogens

In addition to their critical role in the control of puberty, reproduction, growth, and development, estrogens act both centrally and peripherally to regulate energy balance (96, 97). Physiological, pathological, pharmacological, or genetically-induced estrogen deficiency promotes obesity by increasing appetite and reducing energy expenditure, which could be reversed by estrogen replacement (98, 99). Despite that E2 can modulate metabolism by directly acting on peripheral tissues, emerging evidence suggests that the hypothalamus mediates a large part of the actions of E2 on energy balance (100). For instance, estrogens receptors (ERs), including ERα and ERβ, are highly expressed in the hypothalamus (101, 102). ERα is believed to be the major mediator of the effects of estrogens on energy homeostasis. Food intake and body weight are suppressed by central administration of the ERα agonist propylpyrazole triol (PPT), but not by the selective ERβ agonist diarylpropionitrile (DPN) (103, 104). In addition, female mice with a targeted deletion of the ERα gene develop obesity, primarily due to decreased energy expenditure (99). Ablation of ERβ causes no obvious change in body weight under chow condition, but it promotes fat accumulation and improves insulin sensitivity after challenging with high fat diet (105).

Interestingly, estrogens participate in the regulation of food intake and energy expenditure by engaging in different hypothalamic neuronal populations. Disruption of ERα in POMC neurons augments food intake without affecting energy expenditure (106). On the contrary, silencing ERα within the VMH suppresses energy expenditure with no concomitant alterations in feeding behavior. The same phenotype is recapitulated by ablating ERα in SF1 neurons of the VMH (49, 106). More specifically, the decreased energy expenditure in mice lacking ERα in SF-1 neurons is related to impaired BAT thermogenesis as demonstrated by reduced UCP1 levels in BAT. Administration of E2 into the VMH increases energy expenditure by amplifying the sympathetic outflow to BAT and WAT, which is associated with diminished AMPK activity in the VMH (64). Genetic activation of AMPK in the VMH blunts E2-induced weight loss and activation of thermogenesis in BAT and WAT (64), suggesting that E2 promotes BAT thermogenesis and WAT browning through suppressing AMPK in the VMH.

A recent study found that central injection of E2 alleviates ER stress levels in the hypothalamus through decreasing hypothalamic ceramide levels (53). Additionally, blocking ceramide synthesis in E2 deficient rats attenuates ER stress in the VMH and recapitulates the thermogenic effects of central E2 (53). Similarly, pharmacological or genetic inhibition of ER stress in the VMH of ovariectomized (OVX) rats increases BAT temperature and UCP1 expression, which mimics the outcome of E2 supplementation (53). Together, these findings support the notion that E2-induced decrease of ER stress in the VMH contributes to the maintenance of energy balance by modulating BAT thermogenesis. Considering that AMPK inactivation suppresses ER stress within the VMH, which also mediates the effects of central THs on BAT thermogenesis (52), it is possible that the AMPK (VMH)-ER stress-BAT axis might represent a canonical pathway that underlies hormonal and neuronal control of thermogenesis.

Except for ERα and SF-1 neurons, the role of AMPK in other VMH cell types in regulating E2-induced thermogenesis lacks thorough investigation. For example, it has been reported that hypothalamic kisspeptin/neurokinin B/dynorphin (KNDy) neurons are involved in the modulation of body temperature by E2, but whether AMPK in the VMH participates in this process remains to be clarified (107). Moreover, it is noteworthy that the thermogenic effects of E2 are diminished during gestation (108). Although hypothalamic AMPK signaling is inhibited by high circulating E2 levels, pregnant animals exhibit reduced temperature and BAT function (108). These observations raise the hypothesis that pregnancy promotes a state of resistance to the actions of E2, which may partially account for the gestational hyperphagia and fat accumulation to meet the metabolic demands of embryonic development (108). Unraveling the underlying mechanisms of E2 resistance may facilitate the development of new strategies to counteract obesity. Notably, the GLP-1-estrogen conjugate, which is designed to activate estrogen receptors in GLP-1 targeted tissues, has superior efficiency over either of the individual hormones to overcome obesity, hyperglycemia, and dyslipidemia while at the same time prevents hallmark side effects of estrogen such as reproductive toxicity and oncogenicity (109). GLP-1Rs deletion in the brain abrogates the beneficial metabolic consequences of the GLP-1-estrogen conjugate, indicating the CNS is a key action site for this chimera (109). Based on these findings, the use of peptide chimeras appears to be a promising approach in the context of overcoming obesity, but the underlying principles require further elucidation.

### BMP8B

Bone morphogenetic proteins (BMPs) belong to the transforming growth factor β (TGFβ) superfamily and regulate a wide range of physiological processes from embryonic development to tissue homeostasis (110). In recent years, BMPs have been discovered to play a key role in the differentiation and development of BAT as well as in the maintenance of energy balance (110–112). Among the BMPs superfamily, BMP8B is expressed in BAT and the hypothalamus, and is involved in the regulation of BAT function and thermogenesis (113). Central administration of BMP8B increases BAT temperature and the activity of the SNS, resulting in reduced body weight (65). More specifically, intracerebroventricular injection of BMP8B stimulates neuronal activation in both the VMH and LHA (65). Nevertheless, injection of BMP8B into the VMH, rather than the LHA, promotes weight loss and enhances BAT thermogenesis and WAT browning without altering feeding behavior, suggesting the VMH is a direct targeting site for BPM8B (65, 114). Furthermore, the thermogenic action of central BMP8B is AMPK-dependent. BMP8B administration decreases AMPK activity in the VMH, while activation of AMPK within the VMH diminishes BMP8B-induced UCP1 expression in BAT and WAT (65, 114).

Interestingly, central injection of BMP8B stimulates UCP1 expression in brown and white adipocytes in females but not males, indicating that the thermogenic action of central BMP8B is sexually dimorphic (114). In addition, central administration of BMP8B fails to activate the thermogenic program in BAT and WAT in OVX female rats. E2 replacement restores the thermogenic response to BMP8B in OVX rats, further supporting that the presence of E2 is required for BMP8B to fully perform its function on thermogenesis (114). Nevertheless, it is not yet clear how E2 mediates the action of BMP8B, and future work is necessary for clarifying this issue.

Despite BMP8B cannot directly activate neurons in the LHA, central administration of BMP8B increases orexin (OX) levels in the LHA, which is relevant to the inhibition of AMPK within the VMH. Conversely, constitutive activation of AMPK within the VMH reduces BMP8B-induced elevation of OX in the LHA (114). Furthermore, rats pre-treated with SB-334867, an antagonist of OX receptor1 (OX1R), show significantly blunted thermogenic response to BMP8B without affecting AMPK signaling in the VMH, suggesting OX1R signaling is indispensable for the thermogenic actions of BMP8B and is a downstream mediator of AMPK (114). Suppressing glutamatergic signaling in the LHA by deleting GLUT2 (glutamate vesicular transporter 2) abolishes the effects of central BMP8B on BAT thermogenesis and reduces OX levels in the LHA, but has no significant influence on AMPK activity in the VMH, indicating that glutamatergic signaling acts downstream of AMPK to up-regulate OX expression in the LHA (114). Collectively, these results demonstrate that the thermogenic effects of BMP8B are mediated by the inhibition of AMPK in the VMH, the activation of glutamatergic signaling in the LHA, and the subsequent increase of OX levels (114).

### Leptin

Leptin is a circulating hormone secreted by white adipocytes in proportion to fat mass and informs the brain the status of energy storage (115, 116). Leptin exerts its effects on energy balance mainly by acting on the long-form isoform of leptin receptor (LepRb), which is abundantly expressed in the hypothalamus, including the ARH and VMH (117–119). Mice lacking leptin or leptin receptor encoding genes exhibit morbid obesity associated with hyperphagia and low metabolic rate (115, 120). Re-expression of LepRb in the brain reverses obesity and its related metabolic disorders in LepRb null mutant mice, suggesting the metabolic actions of leptin are largely mediated by the central nervous system (121, 122).

LepRb is a member of the class 1 cytokine receptor superfamily that possesses endogenous tyrosine activity (117). The Janus kinase 2/signal transducer and activator of transcription 3 (JAK2/STAT3) pathway is considered as the central mediator of the weight-reducing effects of leptin (123, 124). Central injection of leptin promotes STAT3-dependent transcription of POMC gene, while at the same time inhibiting the expression of AgRP and NPY, thus suppressing appetite and enhancing thermogenesis and energy expenditure (124). Additionally, leptin recruits others signaling networks, such as the phosphatidylinositol 3 kinase/protein kinase B (PI3K/AKT), mammalian target of rapamycin complex 1 (mTORC1) and AMPK pathways to modulate food intake and energy expenditure (60, 125–127). Noticeably, AMPK coordinates with PI3K/AKT and mTORC1 signals to fully facilitate the functions of leptin in the hypothalamus (128, 129). For example, leptin stimulation promotes the phosphorylation of AMPK at ser485 and ser491 in the hypothalamus through the activation of the PI3K-AKT- mTORC1 pathway, which in turn attenuates AMPK activity, leading to reduced food intake (128).

The melanocortin system is believed to play indispensable roles in controlling leptin induced suppression of food intake and body weight (130). Deletion of lepRb in POMC or AgRP neurons results in hyperphagia and obesity (131, 132). Similarly, the VMH has been identified as a key reaction site for leptin, VMH specific SF-1 knockout mice display leptin resistance and are susceptible to diet-induced obesity (133). Leptin directly depolarizes SF-1 neurons in the VMH (134). Ablation of lepRb selectively in SF-1 neurons exaggerates diet-induced obesity, which is accompanied by impaired thermogenesis, indicating lepRb in SF-1 neurons is required for appropriate thermogenic response to overnutrition (134, 135). Furthermore, recent evidence has revealed the involvement of hypothalamic AMPK in mediating the thermogenic actions of leptin (136). Central injection of leptin inhibits hypothalamic AMPK activity and amplifies sympathetic drive to adipose tissues (137). In contrast, constitutive activation of AMPK in the hypothalamus prevents the ability of leptin to increase the sympathetic tone to BAT (60). More specifically, inhibition of the AMPK α2 isoform in the VMH mimics the thermogenic actions of leptin and prevents leptin to further enhance BAT activity, suggesting AMPK within the VMH is at least partially responsible for leptin-stimulated thermogenesis (137).

Additionally, mice lacking protein tyrosine phosphatase 1B (PTP1B), a negative regulator of leptin signaling, in the brain exhibit reduced UCP1 expression in BAT, which is associated with diminished AMPK activity in the hypothalamus (136). Nevertheless, whether AMPK within the VMH contributes to central PTP1B deficiency induced thermogenesis remains to be elucidated, creating and characterizing animal models with PTP1B deletion in the VMH may shed light on this question. Moreover, ablation of the suppressor of cytokine signaling 3 (SOCS3) in SF-1 neurons enhances leptin sensitivity and promotes modest weight loss during lactation, although food intake is not affected (138). However, the role of AMPK in this process warrants further investigation.

## Er Stress in the VMH: Linking AMPK to Thermogenesis?

The ER is a dynamic organ where proteins are matured, assembled and folded (139, 140). Improperly folded proteins are normally delivered to the ER for degradation (139). However, strong and prolonged cellular perturbations may alter ER homeostasis, leading to the accumulation of potentially toxic misfolded proteins and ER stress (141). Evidence accumulated in the past years has revealed a close relationship between ER stress and obesity (141). Particularly, genetic and diet-induced obesity models are associated with elevated ER stress levels in the hypothalamus (142). Central injection of ER stress inducers, such as tunicamycin or thapsigargin, accelerates the development of obesity (52, 143). In contrast, alleviating ER stress by treating obese animals with chemical chaperones, like tauroursodeoxycholic acid (TUDCA) or 4-phenylbutyrate (4-PBA), increases leptin sensitivity and attenuates the risk of obesity (53). Very recently, Contreras and colleagues found that the impaired thermogenesis in BAT and WAT of obese rats is closely related to the elevated ER stress levels in the VMH (51). Intra VMH injection of ceramide triggers ER stress, resulting in an obese phenotype characterized by decreased thermogenic markers in BAT and WAT (51). Inducing ER stress by inhibiting GRP78 in the VMH, but not in the ARH, increases body weight and decreases UCP1 concentrations in BAT (52). Conversely, overexpressing GRP78 in the VMH reduces ER stress, enhancing thermogenesis in BAT and WAT and improving metabolic profiles in obese animals (51, 144), suggesting that ER stress in the VMH plays a critical role in regulating thermogenesis and energy balance.

It is interesting that peripheral signals, such as THs, GLP-1, E2, BMP8B, and leptin, act on the VMH to inhibit AMPK, which subsequently enhances BAT thermogenesis through activating the SNS (16, 52, 64, 66, 114). Both THs and E2 have been reported to suppress AMPK in the VMH and alleviate ER stress in the hypothalamus (52, 53). Inactivation of AMPK within the VMH reduces ER stress levels, whereas constitutive activation of AMPK prevents T3-induced down-regulation of ER stress (52), indicating AMPK acts as an upstream regulator of ER stress in the VMH. Currently, the detailed mechanisms through which AMPK affects ER stress are not fully understood. One acknowledged explanation is that AMPK alters cellular lipid composition by regulating its downstream mediator carnitine palmitoyltransferase 1 (CPT-1), resulting in elevated intracellular ceramide contents, which cause lipotoxicity and trigger the initiation of ER stress (52). In line with this observation, mice lacking CPT-1C display higher hypothalamic ER stress levels and body weight as well as impaired thermogenic response to short-term HFD exposure (145). Given the fact that THs, GLP-1, E2, BMP8B and leptin all stimulate BAT thermogenesis by inhibiting AMPK within the VMH and the close relationship between hypothalamic AMPK and ER stress (16, 52, 64, 66, 114), the VMH AMPK-ER stress-BAT axis may represent a canonical pathway for multiple peripheral signals that act on the VMH to control thermogenesis, although more studies are needed to further testify this hypothesis.

## Targeting AMPK Within the VMH to Counteract Obesity

AMPK is a major energy regulator which exerts opposite actions regarding metabolism in the CNS and the periphery (146–148). On the one side, activation of AMPK promotes fatty acid oxidation and lipolysis in skeletal muscle, and diminishes glucose production in liver, contributing to the maintenance of lipid and glucose homeostasis (149, 150). On the other side, AMPK activation in the hypothalamus augments food intake and suppresses energy expenditure, promoting the development of obesity (146). Therefore, neither systematic activation nor inhibition of AMPK would be a good strategy for the treatment of obesity. In addition, although AMPK has been explored as a pharmacological target for years, the potential cardiac toxicity effects of systematically administrated AMPK activators prevent their clinical application. Therefore, site specific manipulation of AMPK is necessary in order to achieve better outcomes.

Both the VMH and LHA are important areas responsible for the control of food intake and adaptive thermogenesis (151–155). Previous virus tracing experiments have demonstrated that SF-1 neurons in the VMH project to and terminate in the LHA, providing evidence that these two nuclei are anatomically connected (10, 156). On the other hand, the AMPK (VMH)–glutamate- OX (LHA) pathway unravels a molecular basis for the functional interplay between these two major areas for the modulation of BAT thermogenic activity (114). It is of interest to test whether the AMPK (VMH)–glutamate- OX (LHA)-SNS-BAT axis is a universal determinant mechanism for peripheral hormones to regulate BAT thermogenesis (16, 52, 64, 65). Obviously, addressing this question is of considerable significance if we wish to fully understand the hormonal and neuronal control of BAT thermogenesis and may pave the way for developing novel therapies to overcome obesity.

Activation of rat-insulin-promoter-cre (RIP-Cre) neurons in the VMH preferentially promotes the recruitment of beige fat but has no effect on BAT (157). In contrast, inhibiting AMPKα1 activity in SF-1 neurons increases thermogenesis in both BAT and WAT (18). Additionally, central administration of BMP8B increases the sympathetic outflow to BAT but does not alter the sympathetic tone to kidney (114). These findings together indicate that the sympathetic innervations to different organs might be orchestrated by distinct subsets of neurons in the CNS. Notably, obesity is accompanied by elevated sympathetic tone to the cardiovascular system, which is a major contributor to obesity-related hypertension and heart disease (158). Hence, in order to stimulate thermogenesis specifically in adipocytes while circumventing detrimental cardiovascular effects, systematically examining the sympathetic connections between the VMH and peripheral tissues is necessary.

Furthermore, AMPK within the VMH plays a crucial role in the detection of acute hypoglycemia and the initiation of the glucose counter-regulatory response (159–162). Thereby, the potential hypoglycemic risk should be taken into consideration before the application of AMPK inhibitors. Nevertheless, it is noteworthy that the α1, but not the α2, isoform of AMPK within the VMH is mainly responsible for BAT thermogenesis and WAT browning (16, 18, 52). Conversely, the AMPK α2, but not the α1, isoform is a key contributor to the hypoglycemia regulation in the VMH (159–161). In this sense, delicately designed drugs that specifically inhibit the AMPK α1, but not the α2, isoform might be helpful to selectively enhance adaptive thermogenesis and at the same time circumvent the hypoglycemia issue.

Nevertheless, even though AMPK serves as a promising target for obesity in a number of animal models, plenty of difficulties need to be addressed before the clinical application of drugs that modify AMPK in treating human obesity. Firstly, due to the multifaceted actions of AMPK in different organs, site-specific manipulation of AMPK is required but hard to achieve in humans. Besides, the advancement of technology allows for region-selective or even neuron-selective gene manipulations in experimental animals, but targeting specific brain areas in humans remains challenging. In addition, central regulation of thermogenesis requires intact sympathetic innervations to adipocytes. Obesity is often accompanied by impaired sympathetic nerve distributions in fat tissues (163), which may jeopardize the anti-obesity effects of AMPK inhibition in the VMH. Moreover, while the β-AR agonist Mirabegron robustly stimulates glucose uptake in BAT of healthy adult humans (164), administration of a panadrenergic agonist Ephedrine produces minimal effects on BAT activity in obese subjects (165), suggesting the development of β-AR resistance in obesity. Finally, the distribution and regulation of brown and beige adipocytes in humans are not the same as that of rodents. Therefore, whether orchestrating AMPK within the VMH in humans would produce similar beneficial metabolic outcomes waits to be tested.

## Concluding Remarks

**Figure 1:**
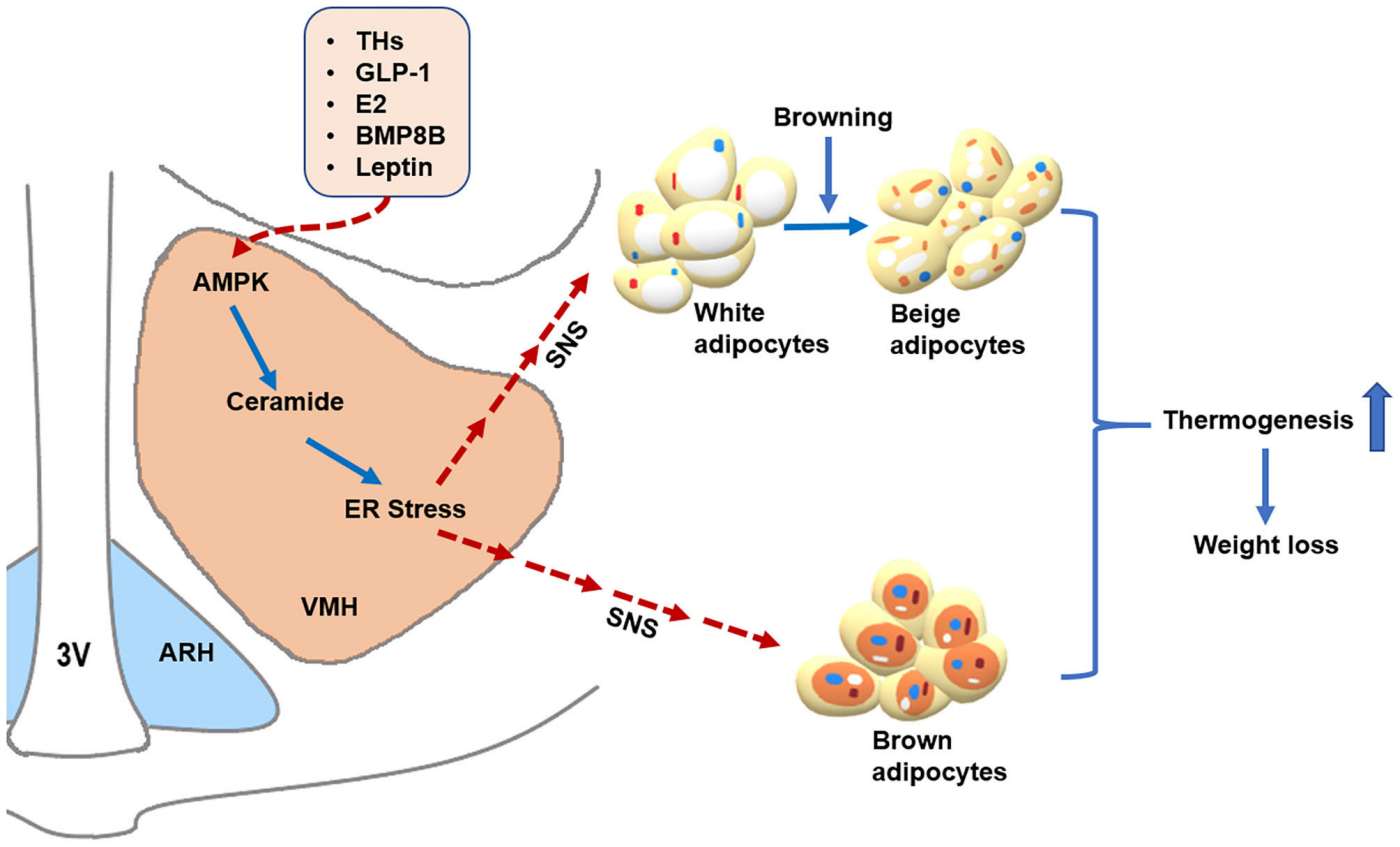
AMPK within the VMH regulates BAT thermogenesis and WAT browning. Peripheral signals, such as THs, GLP-1, E2, BMP8B, and leptin, may act on the VMH to inhibit AMPK, which subsequently promotes BAT thermogenesis and WAT browning through activating the SNS, resulting in body weight loss. Additionally, THs and E2 may decrease ceramide and ER stress levels by suppressing AMPK in the VMH, leading to enhanced thermogenesis via the SNS. Alleviating ER stress in the VMH also increases hypothalamic leptin sensitivity. Whether ceramide and ER stress can mediate the thermogenic effects of GLP-1 and BMP8B warrant further investigation. The solid blue arrows represent activation, the dotted red arrows represent inhibition. 3V: third ventricle. ARH: arcuate nucleus of the hypothalamus. VMH: ventromedial nucleus of the hypothalamus. AMPK: adenosine monophosphate-activated protein kinase. ER stress: endoplasmic reticulum stress. SNS: sympathetic nervous system. THs: thyroid hormones. GLP-1: glucagon-like peptide-1. E2: estrogens. BMP8B: Bone morphogenetic protein 8B.

As summarized in Figure 1, the importance of AMPK within the VMH in regulating thermogenesis is demonstrated by the fact that central THs, GLP-1, E2, BMP8B, and leptin all increase BAT thermogenesis and WAT browning by inhibiting AMPK in the VMH (16, 52, 64–66). Furthermore, ER stress in the VMH mediates the effects of AMPK on thermogenesis (52), suggesting that ER stress is another useful target for obesity. Chemical chaperones, like TUDCA and 4-PBA, are sufficient to reduce hypothalamic ER stress and thereby decreases the risk of obesity (51, 52). More importantly, clinical evidence indicates that some of these chemical chaperones have high safety profiles in humans (166, 167). Thus, designing drugs that act specifically on the VMH to inhibit AMPK or ER stress might represent a promising approach for fighting against obesity.

Currently, there are still some fundamental questions that need to be addressed to fully uncover the role of AMPK in the VMH. First, although recent studies have demonstrated that SF-1 neurons are the key neuronal population which mediates the regulatory effect of AMPK on thermogenesis (18), the involvement of other neuronal populations in thermoregulation is poorly understood. Second, THs and E2 also reduce ER stress in the hypothalamus by inhibiting AMPK in the VMH, which contributes to the thermogenic actions of these hormones (52, 53). It is of significance to explore whether ER stress is a common downstream mediator for the thermogenic effects of THs, GLP-1, E2, BMP8B, and leptin. Third, how different hormones act on the VMH to inhibit AMPK is another question that warrants further investigation. Fourth, a better understanding of how VMH neurons regulate sympathetic outflow to different organs is required for avoiding undesirable side effects.

Overall, considering the critical role of AMPK within the VMH in regulating thermogenesis and the existence of brown and beige adipocytes in adult humans, more investigations are needed to expand our understanding of the neuronal and hormonal control of adaptive thermogenesis and the role of AMPK within the VMH in this process. Addressing these questions may facilitate the development of drugs that are specifically targeted at AMPK within the VMH to enhance thermogenesis and reduce body weight as well as bypass unexpected detrimental effects.

## Author Contributions

HL organized and wrote the manuscript. YX provided constructive comments. FH revised the manuscript. All authors contributed to the article and approved the submitted version.

## Conflict of Interest

The authors declare that the research was conducted in the absence of any commercial or financial relationships that could be construed as a potential conflict of interest.
